# Protective Effect of Free and Bound Polyphenol Extracts from Ginger (*Zingiber officinale* Roscoe) on the Hepatic Antioxidant and Some Carbohydrate Metabolizing Enzymes of Streptozotocin-Induced Diabetic Rats

**DOI:** 10.1155/2013/935486

**Published:** 2013-12-03

**Authors:** Mutiu Idowu Kazeem, Musbau Adewunmi Akanji, Musa Toyin Yakubu, Anofi Omotayo Tom Ashafa

**Affiliations:** ^1^Department of Biochemistry, University of Ilorin, PMB 1515, Ilorin, Nigeria; ^2^Phytomedicine and Phytopharmacology Research Group, Department of Plant Sciences, University of the Free State, Qwaqwa Campus, Phuthaditjhaba, South Africa

## Abstract

This study investigated the hepatoprotective effects of polyphenols from *Zingiber officinale* on streptozotocin-induced diabetic rats by assessing liver antioxidant enzymes, carbohydrate-metabolizing enzymes and liver function indices. Initial oral glucose tolerance test was conducted using 125 mg/kg, 250 mg/kg, and 500 mg/kg body weight of both free and bound polyphenols from *Z. officinale*. 28 day daily oral administration of 500 mg/kg body weight of free and bound polyphenols from *Z. officinale* to streptozotocin-induced (50 mg/kg) diabetic rats significantly reduced (*P* < 0.05) the fasting blood glucose compared to control groups. There was significant increase (*P* < 0.05) in the antioxidant enzymes activities in the animals treated with both polyphenols. Similarly, the polyphenols normalised the activities of some carbohydrate metabolic enzymes (hexokinase and phosphofructokinase) in the liver of the rats treated with it and significantly reduced (*P* < 0.05) the activities of liver function enzymes. The results from the present study have shown that both free and bound polyphenols from *Z. officinale* especially the free polyphenol could ameliorate liver disorders caused by diabetes mellitus in rats. This further validates the use of this species as medicinal herb and spice by the larger population of Nigerians.

## 1. Introduction

Diabetes is a chronic metabolic disorder which has been in existence since time immemorial and affects about 4-5% of the population worldwide [1]. Its complications cause disability in its sufferers leading to frequent hospitalization and huge financial burden [2]. It is a “modern day epidemic” and is given attention as a worldwide public health problem. The number of people suffering from this disease globally is rising on a daily basis with an estimated 366 million people likely to be affected by the year 2030 as against 191 million estimated in 2000 [3]. The management of diabetes mellitus is considered a global problem and successful treatment is yet not available.

Studies have shown that diabetes mellitus is related to oxidative stress, leading to an increased generation of free radicals such as superoxide radical (O_2_^•−^), hydrogen peroxide (H_2_O_2_) and hydroxyl radical (OH^•^) or reduced antioxidant defense mechanism [4, 5]. Effect of oxidative stress in the progression of diabetes mellitus is not only by free radical generation but also due to non-enzymatic protein glycation, impaired antioxidant enzyme system and formation of peroxides [6] which may lead to liver disorder.

Pharmaceutical agents from plants such as polyphenols have been utilised in the treatment of many diseases including diabetes and its complications [7, 8]. Polyphenols are integral part of human diet and are present in plant extracts that have been used in alternative medicine. The antioxidant potential of polyphenols is believed to account in large part for their pharmacological activities [9]. Polyphenols show several pharmacological activities including apoptotic, antidiabetic, antitumor, cardiovascular protection, hepatoprotective, and cell proliferation activities [10].

This study was aimed at evaluating the hepatoprotective effects of polyphenols extracted from *Zingiber officinale* in streptozotocin-induced diabetic rats. Since the medicinal attribute of this species has not been reported in any scientific literature, yet *Z. officinale* is a medicinal herb/spice in Nigeria and may also help in the amelioration of liver damages caused by diabetes.

## 2. Materials and Methods

### 2.1. Plant Material

*Zingiber officinale* was purchased from the Central Spices Market in Mile 12 area, Ketu, Lagos, Nigeria. The identification and authentication of the sample were done by Dr. Kadiri at the Department of Botany of the University of Lagos, Akoka, Lagos, and voucher specimen (LUH 4730) was deposited in the university herbarium.

### 2.2. Experimental Animals

Albino rats were obtained from the Animal House of the Department of Biochemistry, Lagos State University, Ojo, Lagos. All the animals were maintained under laboratory conditions of temperature (22 ± 2°C), humidity (45 ± 5%), and 12 h day: 12 h night cycle and were allowed access to food (standard pellet diet) and water *ad libitum.*

### 2.3. Chemicals

Streptozotocin (STZ) was a product of Alexis Biochemical, San Diego CA (92101), USA, while glibenclamide was a product of Sigma, St. Louis (93101), MO, USA. The assay kit for glucose was obtained from Randox Laboratories, Antrim (BT41), UK. Other chemicals and reagents were of Analar grade, and water used was glass distilled.

### 2.4. Extraction of Free Phenolic Compounds

A known mass (3 kg) of *Zingiber officinale* was crushed in 80% acetone (1 : 2 w/v) using a Waring blender (Waring Commercial, Torrington, CT) for 5 minutes [11]. The sample was homogenized in a Polytron homogenizer (Glen Mills Inc., Clifton, NJ) for 3 minutes. The homogenates were filtered under vacuum using Buchner funnel and Whatman no. 2 filter paper (Whatman PLC, Middlesex, UK). The filtrate was concentrated using a rotary evaporator under vacuum and later freeze-dried in a lyophilizer (Ilshin Lab. Co. Ltd, Seoul, Republic of Korea). The extract was stored frozen at −20°C for 24 h before the commencement of the experiment.

### 2.5. Extraction of Bound Phenolic Compounds

Residue from the free phenolic extraction was drained and hydrolyzed with 2 L of 4 M NaOH for 1 h with constant shaking [11]. The mixture was acidified with concentrated hydrochloric acid to pH 2. The acidified mixture was extracted six times with ethyl acetate by partitioning and the supernatant pooled together and concentrated using rotary evaporator and subsequently freeze-dried using Virtis Bench Top (SP Scientific Series, USA) freeze dryer. The extract was stored frozen at −20°C for 24 h before the commencement of the experiment.

### 2.6. Induction of Diabetes

Rats were fasted for 18 h after which diabetes mellitus (type 2) was induced by single intraperitoneal injection of freshly prepared STZ (50 mg/kg bw) in 0.1 M citrate buffer (pH 4.5) [12]. Diabetes was confirmed in these rats within a period of 7 days. The control animals were administered citrate buffer (pH 4.5). To overcome the initial hypoglycaemic shock, which may occur as a result of the STZ administration, diabetic rats were given 5% glucose solution *ad libitum* for 24 h. Blood was collected from the tail, and the blood glucose level of each rat was determined. Rats with a fasting blood glucose range of 15–20 mmol/L were considered diabetic and included in the study [12].

### 2.7. Oral Glucose Tolerance Test (OGTT)

Twenty-four rats were randomised into 8 groups of 3 animals each. All the rats were fasted overnight (12–14 h) prior to this test. Group 1 was made up of normal rats while the others were streptozotocin-induced diabetic rats. Groups 3, 4, and 5 were administered 125, 250, and 500 mg/kg body weight of free polyphenol from *Z. officinale* while groups 6, 7, and 8 were administered 125, 250, and 500 mg/kg body weight of bound polyphenol from *Z. officinale,* respectively. Forty-five (45) minutes following the various treatment schedules, each rat was administered an oral glucose load (3 g/kg body weight). All rats were tested for blood glucose levels at 45 minutes before the administration of the extracts, 0 minutes (just before the oral administration of glucose load), and 30, 45, 60, and 120 minutes after the glucose load.

### 2.8. Experimental Design

A total of 40 male rats weighing 200 ± 10 g (8 normal; 32 STZ-diabetic rats) were used. The rats were randomised into five groups of eight animals each. Group 1 comprises normal rats administered with vehicle alone (distilled water) and serve as normal control; group 2 consisted of STZ-induced diabetic rats only; groups 3 and 4 comprise STZ-induced diabetic rats administered with 500 mg/kg bw free and bound polyphenol extracts of *Z. officinale*, respectively. Group 5 consisted of STZ-induced diabetic rats administered with glibenclamide (0.6 mg/kg bw). The extract was suspended in distilled water and was orally administered daily for 28 days using orogastric tube. Day 1 was regarded as the first day of treatment with polyphenol extracts or glibenclamide. After 28 days of administration, animals were humanely sacrificed under halothane euthanasia, and blood was collected through cardiac puncture, and serum was separated immediately. The rats were dissected; the liver was excised, freed of surrounding tissues, blotted with clean tissue paper, weighed, and homogenized in ice-cold 0.25 M sucrose solution (1 : 5 w/v). The homogenates were centrifuged at 105 ×g for 15 minutes to obtain the supernatants that were kept frozen overnight at –20°C before being used for the assays.

### 2.9. Biochemical Parameters

#### 2.9.1. Determination of Glucose Concentration

Glucose concentration was estimated by the glucose oxidase method described by Trinder [13].

#### 2.9.2. Determination of Hepatic Antioxidant Enzymes

Catalase (CAT), superoxide dismutase (SOD), and glutathione peroxidase (GPx) activities were determined using the procedure described by Aebi [14], S. Marklund and G. Marklund [15], and Paglia and Valentine [16], respectively. Reduced glutathione (GSH) content was determined according to the method described by Ellman [17].

#### 2.9.3. Determination of Hepatic Carbohydrate Enzymes

Hexokinase, phosphofructokinase, glucose-6-phosphatase, and fructose-1,6-bisphosphatase activities were assayed in the liver by the methods of Brandstrup et al. [18], Castano et al. [19], Hikaru and Toshitsugu [20], and J. M. Gancedo and C. Gancedo [21], respectively. Glycogen content was determined according to the procedure described by Ong and Khoo [22].

#### 2.9.4. Liver Function Test

Alanine aminotransferase (ALT), aspartate aminotransferase (AST), albumin and bilirubin were measured by standard techniques using Reflotron Plus Dry chemistry analyzer (Roche Diagnostics, Mannheim, Germany).

#### 2.9.5. Statistical Analysis

Statistical analysis was performed using GraphPad Prism 5 statistical package (GraphPad Software, USA). The data were analysed by one way analysis of variance (ANOVA) followed by Bonferroni test. All the results were expressed as mean ± SE for 8 rats in each group.

## 3. Results

### 3.1. Effect on Postprandial Blood Glucose

**Figure 1:**
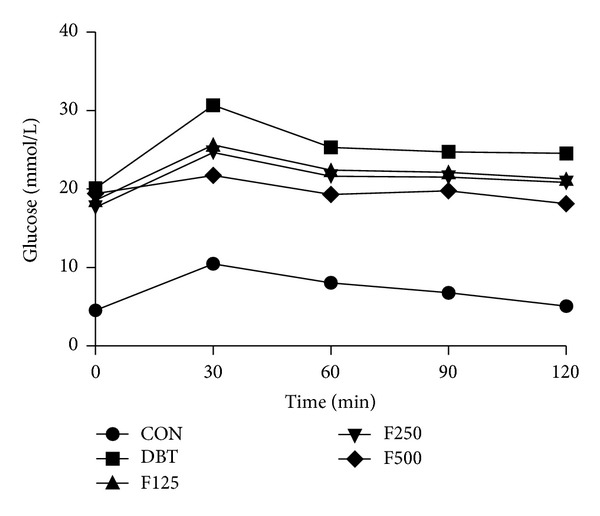
Effect of free polyphenol extract of *Z. officinale* on postprandial blood glucose in streptozotocin-induced diabetic rats. Values are given as mean ± SEM, *n* = 3. CON: normal control, DBT: diabetic control, F125: diabetic rats + 125 mg/kg bw free polyphenol, F250: diabetic rats + 250 mg/kg bw free polyphenol, F500: diabetic rats + 500 mg/kg bw free polyphenol.

**Figure 2:**
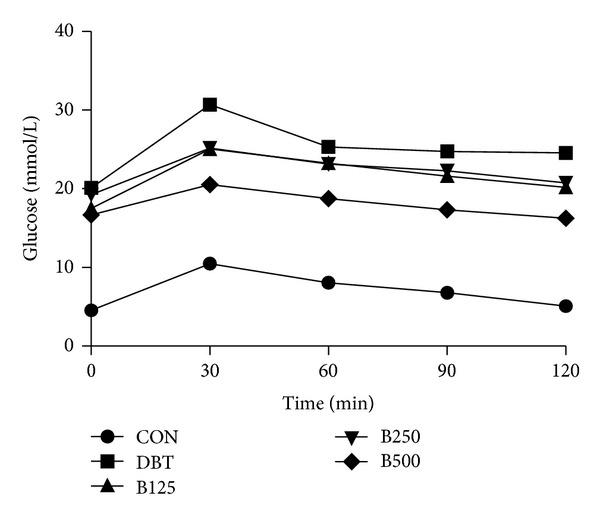
Effect of bound polyphenol extract of *Z. officinale* on postprandial blood glucose in streptozotocin-induced diabetic rats. Values are given as mean ± SEM, *n* = 3. CON: normal control, DBT: diabetic control, B125: diabetic rats + 125 mg/kg bw bound polyphenol, B250: diabetic rats + 250 mg/kg bw bound polyphenol, B500: diabetic rats + 500 mg/kg bw bound polyphenol.

The effects of administration of free and bound polyphenol extracts from *Z. officinale* on postprandial blood glucose of male Wistar rats are presented in Figures 1 and 2. The groups of diabetic rats treated with 500 mg/kg of both free and bound polyphenol extracts of *Z. officinale,* respectively, displayed most significant reduction (*P* < 0.05) at all periods tested in comparison to the diabetic control group. Generally, the reduction in postprandial blood glucose was more pronounced in the free polyphenol treated animals compared to the bound polyphenol treated animals and control groups, respectively.

### 3.2. Effect on Fasting Blood Glucose

The results of fasting blood glucose (FBG) of the polyphenol treated animals during the 28-day experimentation are presented in Table 1. The diabetic control animals had increasing fasting blood glucose throughout the period of the experiment rising from 18.34 on the 1st day to 24.87 mmol/L on the 28th day. The FBG of all groups was significantly different from the normal control (*P* < 0.05). However, the animals treated with *Zingiber officinale* free polyphenol displayed significant reduction (*P* < 0.05) in fasting glucose reaching 12.22 mmol/L from the initial 20.44 mmol/L at the end of the experimental period. Similarly, there was significant reduction in fasting glucose level in the animals treated with bound polyphenol (from 21.80 to 16.56 mmol/L) but not compared to the free polyphenol and glibenclamide treated animals.

### 3.3. Effect on Hepatic Antioxidant Enzymes

The activities of catalase (CAT), superoxide dismutase (SOD), and glutathione peroxidase were significantly decreased (*P* < 0.05) in the diabetic control rats while the reduced glutathione had significant increase (*P* < 0.05) (Table 2). The activities of CAT and SOD in the free polyphenol treated animals were compared favourably with those treated with GBN. Although there were increases in the CAT and SOD activities of the bound polyphenol treated animals but the increase was not comparable to what is obtained in the CAT and SOD of free polyphenol treated animals. Similarly, there was significant increase in the GPx and GSH of GBN treated animals but these enzymes level remained similar in both diabetic control and polyphenol-treated animals.

### 3.4. Effect on Carbohydrate Metabolizing Enzymes

Table 3 shows the effect of administration of polyphenols from *Z. officinale* on the activities of carbohydrate metabolic enzymes in the liver of normal and streptozotocin-induced diabetic rats. There were fluctuations in the activities of hexokinase and phosphofructokinase in all the groups of animals studied but was not significantly different (*P* > 0.05) from one another. The activities of fructose-1,6-bisphosphatase and glucose-6-phosphatase, significantly reduced (*P* < 0.05) in the diabetic control rats compared to the normal control. The 28-day administration of polyphenols especially free polyphenol from *Z. officinale* significantly increased fructose-1,6-bisphosphatase and glucose-6-phosphatase activities in the diabetic rats.

### 3.5. Effect on Glycogen Concentration

**Figure 3:**
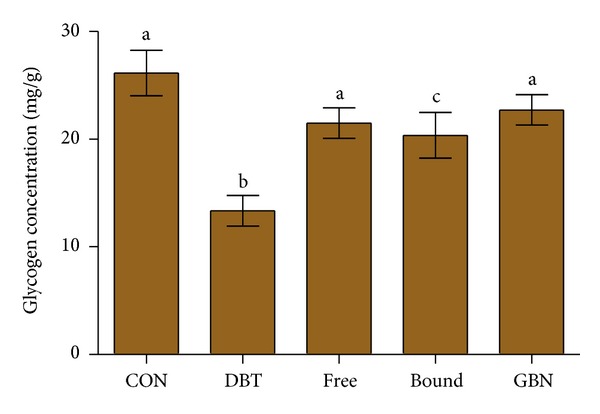
Effect of oral administration of polyphenols from *Z. officinale* on the glycogen concentration in the liver of normal and streptozotocin-induced diabetic rats. Values are mean ± S.E.M. of 8 rats. Bars carrying different superscripts are significantly different (*P* < 0.05). CON: normal control, DBT: diabetic control, Free: diabetic rats + free polyphenol, Bound: diabetic rats + bound polyphenol, GBN: diabetic rats + glibenclamide.

There was a significant reduction (*P* < 0.05) in the glycogen content of diabetic control rats in comparison to the normal control (Figure 3). Diabetic rats treated with polyphenols of *Zingiber officinale* increased the level of glycogen and it is particularly significantly elevated (*P* < 0.05) in the free polyphenol treated animals. The results were compared favourably with the glycogen, concentration of the glibenclamide-treated diabetic rats.

### 3.6. Effect on Liver Function Indices

There was significant elevation (*P* < 0.05) in the activities of both aspartate aminotransferase (AST) and alanine aminotransferase (ALT) in the diabetic untreated animals when compared to the normal control (Table 4). In contrast, there were significant reductions (*P* < 0.05) in these enzyme activities in animals treated with 500 mg/kg polyphenols from *Zingiber officinale* and glibenclamide in comparison to the diabetic control rats. There was no significant difference in the concentration of albumin (ALB) and bilirubin (BIL) of the animals in all groups studied.

## 4. Discussion

The choice of 500 mg/kg body weight of both free and bound polyphenol extracts of *Zingiber officinale* used in the 28-day treatment of diabetic rats in this study was predicated upon the most effective reduction of postprandial blood glucose of diabetic rats following single dose of oral administration in diabetic rats within two hours (oral glucose tolerance test). Though, 125 mg/kg and 250 mg/kg of the polyphenols also decreased postprandial blood glucose but were not comparable to the reduction elicited by the 500 mg/kg dosage (Figures 1 and 2).

Antioxidant enzymes (SOD, CAT, and GPx) play important role in the maintenance of physiological concentrations of oxygen and hydrogen peroxide by enhancing the dismutation of oxygen radicals and mopping up organic peroxides generated from exposure to STZ [23]. The data generated from the present study indicated that STZ-induced diabetes disrupted the activities of hepatic antioxidant enzymes [24]. SOD mop up superoxide radicals by converting them to H_2_O_2_ and oxygen while both CAT and GPx are involved in the elimination of H_2_O_2_ [5]. The observed decrease in the activities of SOD, CAT, and GPx in the liver of diabetic rats may be due to the rise in generation of ROS such as superoxide (O_2_^−^) and hydroxyl (OH^−^) radical [5, 25] by STZ. It may also be that the free radicals generated inactivated the activities of these enzymes [26, 27]. This may be responsible for the insufficiency of antioxidant defences in mitigating ROS mediated damage [6]. However, administration of free polyphenol extract of *Zingiber officinale* reduced the imbalance between the generation of ROS and antioxidant enzymes' activities in diabetic rats. Therefore, treatment with free polyphenol extract of *Zingiber officinale* improved the activities of these antioxidant enzymes and may help to control the production of free radicals in sufferers of diabetes.

Glucose-6-phosphatase (G-6-P) is an important enzyme in the last step of gluconeogenesis and glycogenolysis where it catalyzes the hydrolysis of glucose-6-phosphate to glucose. Glucose is transported out of the liver to increase blood glucose concentration. Physiologically, insulin slows down hepatic glucose production by reducing glucose-6-phosphatase and fructose-1,6-bisphosphatase activities [28, 29]. The hepatic gluconeogenic enzymes, glucose-6-phosphatase, and fructose-1,6-bisphosphatase were elevated significantly in diabetic rats. This may be due to the increased synthesis of the enzymes contributing to the rise in glucose production during diabetes by the liver [30]. The administration of free polyphenols from *Zingiber officinale* normalizes the activities of these enzymes, and this is comparable to both the normal control and glibenclamide-treated diabetic rats. The observed activity may primarily be by modulating the activities of these enzymes, either through the regulation by cyclic adenosine monophosphate (cAMP) or inhibition of glycolysis and gluconeogenesis [30, 31].

Glycogen is the primary intracellular form in which glucose is stored and its levels in various tissues, particularly the liver, are a direct indication of insulin activity as insulin enhances intracellular glycogen deposition by stimulating glycogen synthase and inhibiting glycogen phosphorylase [32]. Because streptozotocin causes selective destruction of *β*-cells in the pancreas, resulting in a noticeable reduction in insulin levels, it implies that glycogen levels in the liver reduce because they depend on insulin for the influx of glucose [33]. Oral administration of free polyphenol extracts of *Z. officinale* significantly improved hepatic glycogen levels of diabetic animals. This is possibly due to the reactivation of the glycogen synthase system as a result of improved insulin secretion following 28-day administration of polyphenol extract to diabetic rats [33]. It may also be due to insulinomimetic activity of the polyphenols giving rise to direct peripheral glucose uptake [34].

Aspartate aminotransferase, alanine aminotransferase, albumin, and bilirubin are considered as part of liver toxicity markers [35]. In streptozotocin-induced diabetic animals, change in the serum enzymes is directly related to alteration in the physiological functions of aspartate aminotransferase, alanine aminotransferase, albumin, and bilirubin [36]. It has been reported that the elevated activities of transaminases under insulin deficiency [37] were responsible for the increased gluconeogenesis and ketogenesis during diabetes. The increase in the activities of these serum enzymes indicated that liver dysfunction might be induced due to diabetes. Previous report has shown that the induction of diabetes in rats with STZ usually leads to necrosis of the liver tissues [38]. Therefore, increase in the activities of serum AST and ALT in diabetic untreated control animals may be due to the leakage of these enzymes from the liver cytosol into the blood stream [39] which is a pointer to the hepatotoxic effect of STZ. Conversely, treatment of the diabetic rats with free and bound polyphenol extracts of *Zingiber officinale* caused reduction in the activity of these enzymes when compared to the diabetic control group and consequently alleviated liver damage caused by STZ-induced diabetes [40].

The difference in the activities of both free and bound polyphenol extracts of *Z. officinale* may not be unconnected to the difference in their structures and compositions. Free polyphenols occur as phenolic acids and flavonoids. They are freely available and more readily absorbed, and, thus, exert beneficial bioactivities in early digestion [41]. Bound polyphenolic compounds, on the other hand, are present as a component of plant cell walls. They are present as monomeric, dimeric, or oligomeric compounds, which are esterified to the cell wall. Bound phytochemicals may not be digested by human enzymes and could survive stomach and intestinal digestion to reach the colon and be digested by bacteria flora releasing phytochemicals with health benefits [11, 42]. Therefore, the potent antioxidant and antidiabetic activities observed in the free polyphenol treated animals may be due to its reported beneficial bioactivity coupled with the ease of digestibility that made it to permeate into the blood system of the treated animals.

## 5. Conclusion

It can be concluded from this study that polyphenols from *Zingiber officinale* offer protection to the liver of diabetic rats. However, free polyphenols of this plant elicited better effect possibly due to the fact that they are freely available and more readily absorbed and exert beneficial bioactivities in early digestion. This study supports the ethnobotanical usage of *Zingiber officinale* rhizome in the treatment of diabetes and its associated complications.

## Figures and Tables

**Table 1:** Effect of administration of polyphenols from *Z. officinale* on the fasting blood glucose of normal and streptozotocin-diabetic rats.

Group	Glucose (mmol/L)		
	Day 1	Day 14	Day 28
Normal control	4.51 ± 0.18^a^	4.44 ± 0.20^a^	4.78 ± 0.36^a^
Diabetic control	18.34 ± 0.47^b^	21.8 ± 0.67^b^	24.87 ± 1.13^b^
Diabetic + free polyphenol	20.44 ± 0.72^b^	21.38 ± 0.95^b^	12.22 ± 1.63^d^
Diabetic + bound polyphenol	21.8 ± 0.67^b^	22.46 ± 0.91^b^	16.56 ± 0.95^c^
Diabetic + glibenclamide	20.48 ± 0.83^b^	17.56 ± 0.39^c^	9.22 ± 0.78^d^

Values are mean ± S.E.M. of 8 rats per group. Test values down the vertical columns carrying different superscripts for each day are significantly different (*P* < 0.05).

**Table 2:** Effect of administration of polyphenols from *Z. officinale* on antioxidant enzymes' activities in the liver of normal and streptozotocin-diabetic rats.

Group	U/mg protein			
	CAT	SOD	GPx	GSH
Normal control	65.20 ± 2.89^a^	33.60 ± 1.73^a^	51.63 ± 2.75^a^	16.21 ± 1.16^a^
Diabetic control	11.25 ± 1.16^b^	7.83 ± 1.21^b^	19.63 ±1.24^b^	28.74 ± 2.67^b^
Diabetic + free	34.56 ± 2.31^c^	24.35 ± 2.17^c^	25.47 ± 1.68^b^	23.37 ± 1.19^b^
Diabetic + bound	21.27 ± 1.83^d^	18.53 ± 1.17^d^	23.75 ± 2.76^b^	25.46 ± 1.70^b^
Diabetic + GBN	40.42 ± 1.76^c^	25.45 ± 1.29^c^	35.20 ± 1.79^c^	20.26 ± 1.12^c^

Values are mean ± S.E.M. of 8 rats. Test values down the vertical columns carrying different superscripts for each parameter are significantly different (*P* < 0.05). CAT: catalase, SOD: superoxide dismutase, GPx: glutathione peroxidase, GSH: reduced glutathione.

**Table 3:** Effect of administration of polyphenols from *Z. officinale* on carbohydrate metabolic enzymes' activities in the liver of normal and streptozotocin-diabetic rats.

Group	U/mg protein			
	HK	PFK	F-1,6-BP	G-6-P
Normal control	2.95 ± 0.03^a^	3.81 ± 0.06^a^	3.56 ± 0.58^a^	10.50 ± 1.16^a^
Diabetic control	0.93 ± 0.36^a^	2.56 ± 0.23^a^	8.42 ±1.16^b^	25.62 ± 2.89^b^
Diabetic + free	2.55 ± 0.23^a^	3.34 ± 0.17^a^	4.23 ± 0.29^a^	13.25 ± 1.16^a^
Diabetic + bound	2.17 ± 0.29^a^	2.86 ± 0.58^a^	5.96 ± 0.58^c^	15.63 ± 1.73^a^
Diabetic + GBN	2.68 ± 0.21^a^	3.69 ± 0.17^a^	4.06 ± 0.51^a^	12.36 ± 2.05^a^

Values are mean ± S.E.M. of 8 rats. Test values down the vertical columns carrying different superscripts for each parameter are significantly different (*P* < 0.05). HK: hexokinase, PFK: phosphofructokinase, F-1,6-BP: fructose-1,6-biphosphatase, G-6-P: glucose-6-phosphatase.

**Table 4:** Effect of administration of polyphenols from *Z. officinale* on liver function parameters of normal and streptozotocin-diabetic rats.

Group	Liver function			
	AST (U/L)	ALT (U/L)	ALB (mg/dL)	BIL (mg/dL)
Normal control	67.2 ± 4.67^a^	131.1 ± 9.44^a^	5.29 ± 1.10^a^	3.30 ± 0.54^a^
Diabetic control	191.4 ± 22.94^b^	251.4 ± 36.96^b^	5.84 ± 1.50^a^	4.84 ± 0.53^a^
Diabetic + free	95.60 ± 15.91^c^	156.6 ± 13.20^c^	5.04 ± 0.93^a^	3.10 ± 0.49^a^
Diabetic + bound	121.0 ± 3.54^d^	181.2 ± 7.87^d^	6.27 ±0.71^a^	4.15 ± 0.72^a^
Diabetic + GBN	83.3 ± 5.54^c^	136.5 ± 11.92^a^	4.89 ± 0.87^a^	2.31 ± 0.60^a^

Values are mean ± S.E.M. of 8 rats per group. Test values down the vertical columns carrying different superscripts for each parameter are significantly different (*P* < 0.05). AST: aspartate aminotransferase, ALT: alanine aminotransferase, ALB: albumin, BIL: bilirubin.

## References

[1] Koyuturk M, Ozsoy-Sacan O, Bolkent S, Yanardag R (2005). Effect of glurenorm on immunohistochemical changes in pancreatic *β* cells of rats in experimental diabetes. *Indian Journal of Experimental Biology*.

[2] Nagappa AN, Thakurdesai PA, Rao NV, Singh J (2003). Antidiabetic activity of *Terminalia catappa* Linn fruits. *Journal of Ethnopharmacology*.

[3] Wild S, Roglic G, Green A, Sicree R, King H (2004). Global prevalence of diabetes: estimates for the year 2000 and projections for 2030. *Diabetes Care*.

[4] Rahimi R, Nikfar S, Larijani B, Abdollahi M (2005). A review on the role of antioxidants in the management of diabetes and its complications. *Biomedicine and Pharmacotherapy*.

[5] Vincent AM, Russell JW, Low P, Feldman EL (2004). Oxidative stress in the pathogenesis of diabetic neuropathy. *Endocrine Reviews*.

[6] Pari L, Latha M (2005). Antidiabetic effect of Scoparia dulcis: effect on lipid peroxidation in streptozotocin diabetes. *General Physiology and Biophysics*.

[7] Pałgan K, Drewa G (1994). Flavonoids in medicine. *Postepy Higieny i Medycyny Doswiadczalnej*.

[8] Machha A, Mustafa MR (2005). Chronic treatment with flavonoids prevents endothelial dysfunction in spontaneously hypertensive rat aorta. *Journal of Cardiovascular Pharmacology*.

[9] Laight DW, Carrier MJ, Änggård EE (2000). Antioxidants, diabetes and endothelial dysfunction. *Cardiovascular Research*.

[10] Han X, Shen T, Lou H (2007). Dietary polyphenols and their biological significance. *International Journal of Molecular Sciences*.

[11] Chu Y-F, Sun J, Wu X, Liu RH (2002). Antioxidant and antiproliferative activities of common vegetables. *Journal of Agricultural and Food Chemistry*.

[12] Eliza J, Daisy P, Ignacimuthu S, Duraipandiyan V (2009). Antidiabetic and antilipidemic effect of eremanthin from *Costus speciosus* (Koen.) Sm., in STZ-induced diabetic rats. *Chemico-Biological Interactions*.

[13] Trinder P (1969). Determination of glucose in blood using glucose oxidase with an alternative oxygen acceptor. *Annals of Clinical Biochemistry*.

[14] Aebi H, Bergmeyer HU (1974). Catalase. *Methods of Enzymatic Analyses*.

[15] Marklund S, Marklund G (1974). Involvement of the superoxide anion radical in the autoxidation of pyrogallol and a convenient assay for superoxide dismutase. *European Journal of Biochemistry*.

[16] Paglia DE, Valentine WN (1967). Studies on the quantitative and qualitative characterization of erythrocyte glutathione peroxidase. *The Journal of Laboratory and Clinical Medicine*.

[17] Ellman GL (1959). Tissue sulfhydryl groups. *Archives of Biochemistry and Biophysics*.

[18] Brandstrup N, Kirk JE, Bruni C (1969). The hexokinase and phosphoglucoisomerase activities of aortic and pulmonary artery tissue in individuals of various ages. *Journal of Gerontology*.

[19] Castaño JG, Nieto A, Felíu JE (1979). Inactivation of phosphofructokinase by glucagon in rat hepatocytes. *Journal of Biological Chemistry*.

[20] Hikaru K, Toshitsugu O (1959). Pathological occurrence of glucose-6-phosphatase in serum in liver diseases. *Clinica Chimica Acta*.

[21] Gancedo JM, Gancedo C (1971). Fructose-1,6-diphosphatase, phosphofructokinase and glucose-6-phosphate dehydrogenase from fermenting and non fermenting yeasts. *Archiv für Mikrobiologie*.

[22] Ong KC, Khoo H-E (2000). Effects of myricetin on glycemia and glycogen metabolism in diabetic rats. *Life Sciences*.

[23] Pari L, Latha M (2004). Protective role of *Scoparia dulcis* plant extract on brain antioxidant status and lipidperoxidation in STZ diabetic male Wistar rats. *BMC Complementary and Alternative Medicine*.

[24] Lee JM, Okumura MJ, Davis MM, Herman WH, Gurney JG (2006). Prevalence and determinants of insulin resistance among U.S. adolescents: a population-based study. *Diabetes Care*.

[25] Kaleem M, Asif M, Ahmed QU, Bano B (2006). Antidiabetic and antioxidant activity of *Annona squamosa* extract in streptozotocin-induced diabetic rats. *Singapore Medical Journal*.

[26] Soon YY, Tan BKH (2002). Evaluation of the hypoglycemic and anti-oxidant activities of *Morinda officinalis* in streptozotocin-induced diabetic rats. *Singapore Medical Journal*.

[27] Ravi K, Ramachandran B, Subramanian S (2004). Protective effect of *Eugenia jambolana* seed kernel on tissue antioxidants in streptozotocin-induced diabetic rats. *Biological and Pharmaceutical Bulletin*.

[28] Wiernsperger NF, Bouskela E (2003). Microcirculation in insulin resistance and diabetes: more than just a complication. *Diabetes and Metabolism*.

[29] Chen R, Meseck M, McEvoy RC, Woo SLC (2000). Glucose-stimulated and self-limiting insulin production by glucose 6-phosphatase promoter driven insulin expression in hepatoma cells. *Gene Therapy*.

[30] Susztak K, Raff AC, Schiffer M, Böttinger EP (2006). Glucose-induced reactive oxygen species cause apoptosis of podocytes and podocyte depletion at the onset of diabetic nephropathy. *Diabetes*.

[31] Baquer NZ, Gupta D, Raju J (1998). Regulation of metabolic pathways in liver and kidney during experimental diabetes: effects of antidiabetic compounds. *Indian Journal of Clinical Biochemistry*.

[32] Grover JK, Vats V, Rathi SS (2000). Anti-hyperglycemic effect of *Eugenia jambolana* and *Tinospora cordifolia* in experimental diabetes and their effects on key metabolic enzymes involved in carbohydrate metabolism. *Journal of Ethnopharmacology*.

[33] Bansal R, Ahmad N, Kidwai JR (1981). Effects of oral administration of *Eugenia jambolana* seeds and chloropropamide on blood glucose level and pancreatic cathepsin B in rat. *Indian Journal of Biochemistry and Biophysics*.

[34] Lolitkar MM, Rao MRR (1996). Pharmacology of a hypoglycaemic principle isolated from the fruits of *Eugenia jambolana* Linn. *Indian Journal of Pharmacy*.

[35] Mori DM, Baviera AM, de Oliveira Ramalho LT, Vendramini RC, Brunetti IL, Pepato MT (2003). Temporal response pattern of biochemical analytes in experimental diabetes. *Biotechnology and Applied Biochemistry*.

[36] Asayama K, Nakane I, Uchida N, Hayashibe H, Dobashi K, Nakazawa S (1994). Serum antioxidant status in streptozotocin-induced diabetic rat. *Hormone and Metabolic Research*.

[37] Felig P, Marliss E, Ohman JL, Cahill CF (1970). Plasma amino acid levels in diabetic ketoacidosis. *Diabetes*.

[38] Ohaeri OC (2001). Effect of garlic oil on the levels of various enzymes in the serum and tissue of streptozotocin diabetic rats. *Bioscience Reports*.

[39] Concepcion Navarro M, Montilla MP, Martin A, Jimenez J, Utrilla MP (1993). Free radical scavenger and antihepatotoxic activity of *Rosmarinus tomentosus*. *Planta Medica*.

[40] El-Demerdash FM, Yousef MI, El-Naga NIA (2005). Biochemical study on the hypoglycemic effects of onion and garlic in alloxan-induced diabetic rats. *Food and Chemical Toxicology*.

[41] Oboh G, Rocha JBT (2007). Distribution and antioxidant activity of polyphenols in ripe and unripe tree pepper (*Capsicum pubescens*). *Journal of Food Biochemistry*.

[42] Sun J, Chu Y-F, Wu X, Liu RH (2002). Antioxidant and antiproliferative activities of common fruits. *Journal of Agricultural and Food Chemistry*.

